# Long-term mental health and quality of life in patients after cervical artery dissection

**DOI:** 10.3389/fneur.2026.1726823

**Published:** 2026-09-15

**Authors:** Katrine Sværke, Sophia Engelter, Cilgia Marugg, Josefin E. Kaufmann, Eveline Brunner, Philippe Lyrer, Henrik Gensicke, Valerian L. Altersberger, Annaelle Zietz, Alexandros Polymeris, Elke Berger, Christian Ovesen, Sverre Rosenbaum, Hanne Christensen, Stefan T. Engelter, Christopher Traenka

**Affiliations:** 1 Department of Neurology, Bispebjerg Hospital & University of Copenhagen, Copenhagen, Denmark; 2 Department of Rehabilitation and Neurology, University Department of Geriatric Medicine FELIX PLATTER, University of Basel, Basel, Switzerland; 3 Department of Neurology and Stroke Center, Department of Clinical Research, University Hospital Basel and University of Basel, Basel, Switzerland

**Keywords:** cervical artery dissection, long-term follow up, mental health, quality of life, stroke

## Abstract

**Introduction:**

Most patients with Cervical Artery Dissection (CeAD) experience good long-term functional outcome. Little is known about long-term psychological sequelae in these patients.

**Patients and methods:**

In CeAD-patients, we assessed long-term quality of life (QoL), frequency of increased anxiety levels, post-traumatic stress disorder (PTSD), depression and fatigue and explored if baseline characteristics can identify patients at high risk of developing the aforementioned. We performed long-term follow-up (> = 12 months after baseline) in CeAD-patients with excellent 3-month outcome and no prior psychiatric disorder. Patients were asked to answer established questionnaires.

**Results:**

Long-term follow-up was performed on 89 CeAD-patients at a median of 53 months after baseline. Sixty-five patients had carotid artery dissection and 37 patients had vertebral artery dissection (13 patients had both). At long-term follow-up the threshold for formal diagnosis of anxiety was met by 14.6, and 11.2% met the criteria for PTSD. 29.2% had a score above threshold for significant peri-traumatic dissociation. 19.1% reported depression at any point between baseline and long-term follow-up and 48.3% scored above thresholds for abnormal fatigue. Those who experienced increased levels of anxiety and those who met criteria for PTSD reported reduced QoL. Peritraumatic dissociation at the acute phase of CeAD was associated with increased anxiety levels at long-term follow-up. Occlusion of the dissected artery, tinnitus at baseline and prior smoking were associated with PTSD at long-term follow-up.

**Discussion:**

Prognostic importance of the experience of peritraumatic dissociation as well as occlusion of the dissected artery requires further research. Further prospective and interventional studies in treatment of psychological sequela in CeAD-patients are warranted.

**Conclusion:**

CeAD-patients seem to have a high long-term risk of developing anxiety, depression or PTSD, which was accompanied by decreased QoL. These findings are exploratory and should be further confirmed.

## Introduction

### Background

Cervical Artery Dissection (CeAD) is the leading cause of ischemic stroke in young adults (<50 years) (1). Most patients with CeAD experience a good physical and functional long-term outcome as measured by the modified Rankin Scale (mRS)-score with low rates of delayed strokes and recurrent dissections beyond 3 months (2–5). Limited evidence indicates that outcomes are not nearly as positive when psychological sequela and long-term quality of life (QoL) are considered (6–8). Beyond potential physical impairments after CeAD associated stroke, psychological sequelae could have important impact on social participation and professional life in these young patients. As such, there is a need for further knowledge on long-term QoL after CeAD, including measures of mental health and psychosocial aspects.

The aim of this study was (i) to assess long-term QoL, frequency of increased anxiety levels, post-traumatic stress disorder (PTSD), depression, fatigue and cognitive complaints in CeAD-patients and (ii) to explore if any baseline variables can identify patients with CeAD being at high risk of developing PTSD or anxiety.

## Methods

This study is a long-term follow-up cohort study of patients with CeAD with excellent 3-month recovery. This study is reported using the STROBE guidelines for reporting cohort studies (9).

### Study design, setting, and population

In this single-center, exploratory study, all consecutive patients from the CeAD-database of the Stroke Center of the University Hospital Basel who met the following criteria were included: (i) excellent 3-month outcome defined by a 3-month mRS of 0–1 (ii) long-term follow-up-data available for ≥ 12 month after CeAD-onset, (iii) participation in a systematic and standardized assessment of mental health issues after CeAD. Patients with a history of any psychiatric disorder (formal diagnosis, either self-reported by potentially eligible participants or registered in medical history) were excluded. Participants were identified through a prospective CeAD registry comprising detailed sets of baseline variables (10), which could be analyzed alongside the long-term follow-up data.

As in previous works (11, 12), CeAD (all extracranial) diagnosis was verified by magnetic resonance arterial imaging applying the following, widely accepted diagnostic criteria (presence of at least one): mural hematoma, aneurysmal dilatation, long tapering stenosis, intimal flap, double lumen, or occlusion ≥2 cm above the carotid bifurcation revealing an aneurysmal dilatation or a long tapering stenosis after recanalization in a cervical artery (1, 13).

Long-term follow-up was performed at least 12 months after index CeAD either during routine outpatient clinical follow-up visits or after patient contact by the local investigators by post, telephone, or e-mail. If performed during clinical visits, participants were allowed to fill out pre-defined, structured questionnaires either at the visit or at a later timepoint and send the completed questionnaires back to the study center. All participants filled in the questionnaires once as part of the long-term follow-up visit. If performed after postal, telephone or e-mail contact, participants received the questionnaires by post and sent the completed questionnaires back to the study center. Reminders were sent out to patients who did not initially respond.

### Variables and measurements

Baseline variables were extracted as described in a previous work (10). The below listed measurements were all performed at long-term follow-up:

PTSD; (i) Peritraumatic Dissociative Experience Questionnaire (PDEQ-10) assessed at long-term follow-up, with patients asked to report their experience during the index CeAD (14). The PDEQ-10 is a self-report questionnaire which assesses the emotional and physiological distress experienced during and/or immediately after a traumatic event (14). The PDEQ-10 provides a score between 10 (no dissociation) and 50 (severe dissociation) based on 10 questions with a 1–5 likert scale. Study uses a cut-off score of 23 for significant dissociation, as this cut-off has been shown to be associated with the development and severity of PTSD (14). Patients were specifically asked to fill out the PDEQ regarding their experience during the index event. (ii) Post-traumatic Stress diagnostic Scale (PDS) at long-term follow-up. The PDS is a self-reported measure of post-traumatic stress disorder (PTSD) based on DSM-IV criteria for PTSD and includes an assessment of both the presence of PTSD and the severity of symptoms (15). PTSD was recorded as present in patients who met all the criteria for PTSD on the PDS-scale according to the scoring manual.

Anxiety; (i) Patients were asked if they felt more anxious after, compared to before the index CeAD. (ii) Hospital Anxiety and Depression Scale (HADS) at long-term follow-up: HADS is a 14-item self-report questionnaire, which is used to identify presence and severity of anxiety and depression in patients (16). A score of 8 or above is used as a general threshold for probable anxiety or depression, 21 is the maximum possible score for both anxiety and depression (16).

Depression; (i) Patients were asked about any history of self-reported depression before the index CeAD. (ii) Patients were asked, if they were diagnosed with depression at any point between the index event and long-term follow-up. (iii) Diagnosis based on HADS at long-term follow-up following the same method and threshold as described above for anxiety.

Quality of Life; Short Form Survery-36 (SF-36): the SF-36 is a QoL-measure across eight domains with a maximum of a 100 points for each domain, with higher scores indicating a higher quality of life. SF-36 was used to incorporate a broad measure of various physical and mental QOL-domains in the cohort (17). Short Form Survery-36 (SF-36): the SF-36 is a QoL-measure across eight domains: Physical functioning, bodily pain, role limitations due to physical health problems, role limitations due to personal or emotional problems, emotional well-being/mental health, social functioning, energy/fatigue, and general health perceptions (17). The SF-36 data collected in this study compare patients with PTSD and anxiety after CeAD to patients without these symptoms after CeAD. Secondarily, the scores for patients in this population are visually represented along with normative SF-36 data from 9,332 healthy, age-matched (45 to 54 years) British adults averaged across the norms for males and females (18).

Fatigue; Checklist Individual Strength (CIS): CIS is a 20-item self-report fatigue questionnaire with a maximum score of 140 (higher scores indicating higher levels of fatigue) to discriminate healthy individuals from individuals experiencing severe mental fatigue (19). This study has used a cut-off score of 35 for severe fatigue, as recommended by earlier research (19). CIS was used to assess the presence of severe mental fatigue as well as to explore the relationship between mental fatigue and heightened anxiety levels and PTSD in the cohort.

In addition, the following outcome variables were assessed at long-term follow-up: (i) returning to work after the index event; (ii) change in job hours and physical activity compared to before index CeAD; (iii) participants’ subjective health-score at long-term follow-up (0 to 100); (iv) self-rated current mRS score; (v) if patients had any severe headaches within the last year; (vi) Which medical risk-factors were present; (vii) If participants currently smoked; (viii) any ischemic events in close relatives.

### Statistical methods

Categorical data were presented using counts and proportions, and analyzed using the chi-square test, except for tables with expected cell counts <5, where Fisher’s exact test was used. Continuous/ordinal data were presented using either mean and standard deviation or median and interquartile range (IQR) as appropriate. Continuous/ordinal data were analyzed using an independent *t*-test. Due to the limited sample size, multivariable logistic regression analyses were considered at high risk of unstable effect estimates. Preliminary exploratory analyses yielded inflated odds ratio, suggesting insufficient model stability. Therefore, associations were explored using univariable analyses, and findings should be interpreted cautiously. Corrections for multiple comparisons were not performed, due to the exploratory nature of the study.

As some baseline variables were not obtained for all participants in the long-term follow-up cohort, the results section states for how many participants any given baseline score/value was not obtained.

Because questionnaires were sent by post to patients who filled them out themselves, a question was occasionally skipped by the participant in an otherwise completed questionnaire. In these cases, the missing score was replaced with an average score calculated from the remaining questions within the same questionnaire. As such, item-level imputation was performed using the participant’s own mean for that questionnaire, group means were not used. The missing responses were: SF-36: One missing response on question 3a, HADS: One missing response on question 6, PDEQ: One missing response on question 3. The three missing responses were from three different participants. Sensitivity analyses were performed by rerunning the analyses excluding these three cases. The sensitivity analyses found no significant changes on any of the questionnaires with missing responses. Statistical analyses were performed using SPSS version 29.0 (IBM Corp., Armonk, NY, USA).

## Results

A total of 89 patients with prior CeAD were eligible for inclusion in the analysis (see Figure 1). Baseline characteristics are presented in Table 1, from which it is also evident that 65 patients had carotid artery dissection and 37 patients had vertebral artery dissection (13 patients had both). Long-term follow-up was performed at a median of 53 months (5.8 years) after index CeAD (range: 15 to 351 months, mean: 69). See Figure 2 for bar chart of distribution of long-term follow-up time. Median (IQR) mRS at long-term follow-up was 0 (IQR: 0–0). At the long-term follow-up visit recurrent stroke had occurred in 10 participants (11.2%) and recurrent CeAD had been diagnosed in 5 (5.6%).

**Figure 1:**
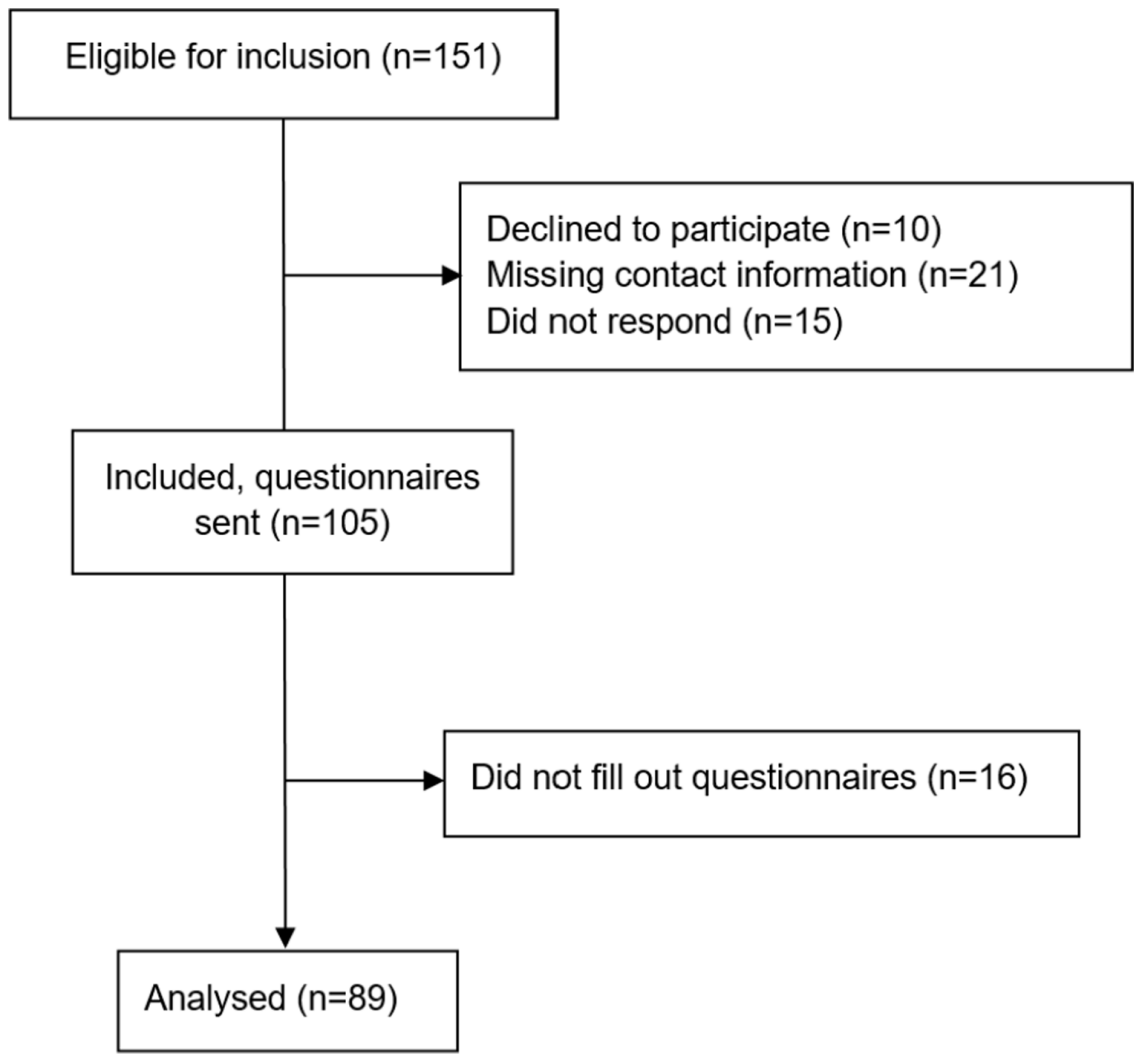
Participant flow diagram.

**Table 1:** Baseline characteristics, outcomes at 3 months and outcomes at long term follow-up (*n* = 89).

Baseline characteristics	
Age at symptom onset	45 (10.4) years
Male gender	50 (56.2%)
Formal education	15.1 (3.6) years
mRS before index event	0 (0 to 0)
NIHSS on admission	0 (0 to 1)
Affected artery (some participants had multiple CeAD)	
Carotid artery dissection	65 (73%)
Vertebral artery dissection	37 (41.6%)
Number of affected arteries	
1	76
2	12
3	1
Ischemic symptoms at index admission	
Ischemic stroke	39 (43.8%)
TIA	12 (13.5%)
Monocular blindness	2 (2.2%)
Local symptoms at index admission	
Headache	66 (74.2%)
Cervical pain	54 (60.7%)
Cranial nerve palsy	21 (23.6%)
Horner’s syndrome	32 (36%)
Tinnitus	21 (23.6%)
Risk factors on admission	
Hypertension	31 (34.8%)
Hypercholesterolemia	12 (13.5%)
Diabetes	1 (1.1%)
History of smoking	17 (19.1%)
History of migraine with aura	6 (6.7%)
History of migraine without aura	16 (18%)
Mechanical trigger event before index event	19 (21.3%)
Prior infection before index event (within 4 weeks)	13 (14.6%)
History of depression before index event	14 (15.7%)
Significant dissociation on admission on the PDEQ	26 (29.2%)
Outcomes at long term follow-up	
Time between index event and follow-up	Median: 53 months (range: 15–351 months)
mRS at follow-up	
Excellent outcome (mRS 0–1)	81 (91%)
Unfavorable outcome (mRS 2–5)	5 (5.6%)
Outcome status unknown	3 (3.4%)
Stroke or new dissection between index event and follow-up	
Any stroke	10 (11.2%)
New dissection	5 (5.6%)
Subjective health from 0 to 100	Mean: 85.8 (SD: 14.8)
Back to job after dissection	80 (89.9%)
Weekly hours of work	
Before dissection	39.6 (12.8) hours
After dissection	36.2 (13.2) hours
Changes in physical activity due to dissection	
Has reduced certain physical activities	15 (16.9%)
Has avoided certain physical activities	19 (21.3%)
Has both reduced and avoided certain physical activities	5 (5.6%)
Has enhanced certain physical activities	6 (6.7%)
No changes due to dissection	41 (46.1%)
Score not obtained	3 (3.4%)
Weekly hours of exercise	
Before dissection	Mean: 8.8 (SD: 10.8) hours
After dissection	Mean: 8.5 (SD: 10.6) hours
Severe headaches within the last year	31 (34.8%)

Data are presented as count (percent), mean (standard deviation) or median (interquartile range) as appropriate. NIHSS, National Institute of Health Stroke Scale; mRS, Modified Rankin Scale; TIA, Transient ischaemic attack.

**Figure 2:**
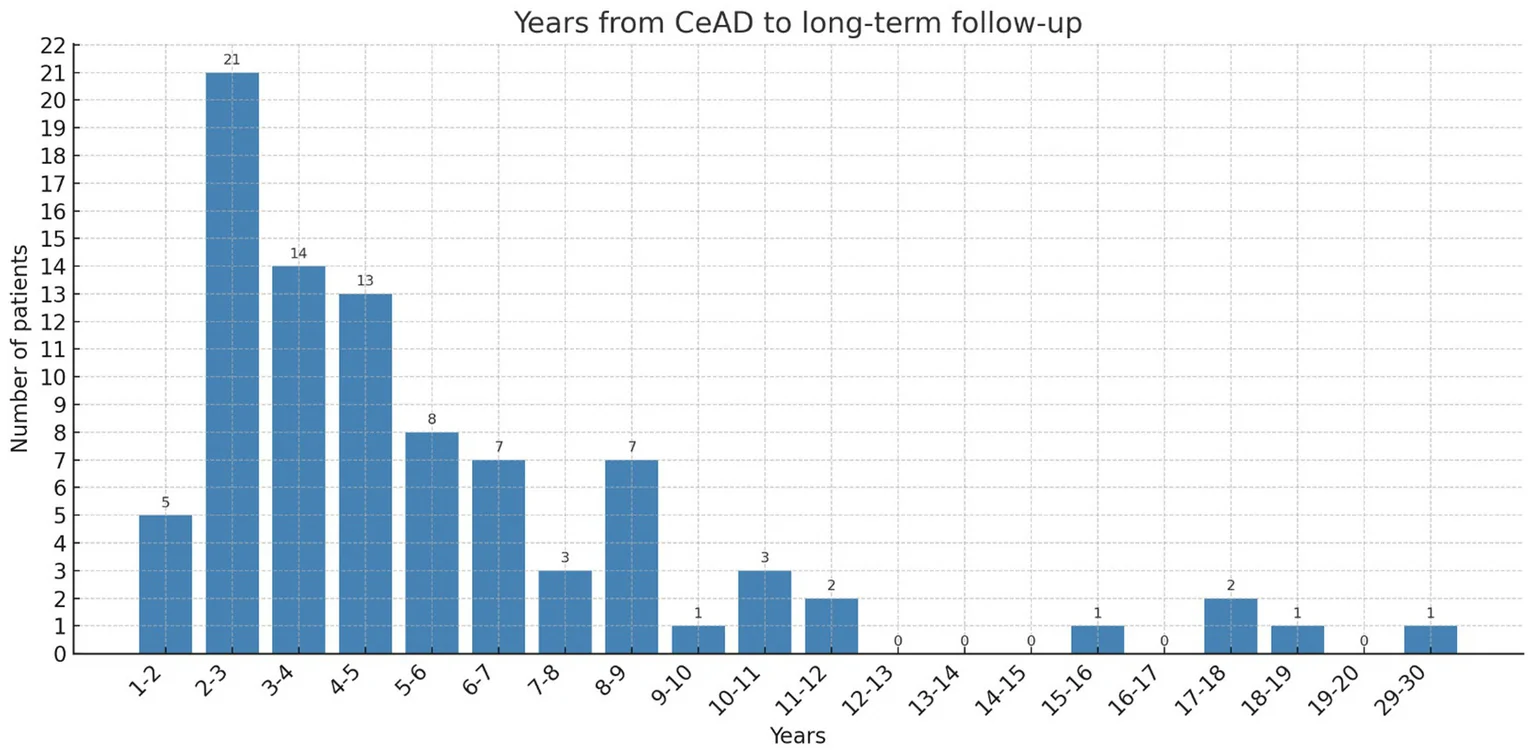
Years from CeAD to long-term follow-up.

### PTSD at long-term follow-up

Twenty-six participants (29.2% out of 89) had a score above threshold for significant dissociation at admission on the PDEQ (Table 1), and 10 participants (11.2%) met the criteria for PTSD on the PDS (Table 2). Participants who met the criteria for PTSD on the PDS at long-term follow-up were more likely to have an occlusion of dissected artery (6[60%] versus 14[18%]), tinnitus at admission (5[50%] versus 16[20%]) and smoking at admission (6[60%] versus 11[14%]) (Table 3). In addition, evidence was observed that participants who met the criteria for PTSD on the PDS at long-term follow-up had worse QoL-scores on four out of eight domains on the SF-36 subdomains: energy/fatigue, emotional well-being, social functioning and general health (Table 3). An independent *t*-test was performed as a sensitivity analysis to explore if there were significant differences in PTSD occurrence between patients who participated in the long-term follow-up study more than 5 years after CeAD (38 patients) or less than 5 years after CeAD (51 patients). No significant differences were found (*p* = 0.625).

**Table 2:** Mental health at long term follow-up (*n* = 89).

Feeling more anxious after the dissection	Yes: 32 (36%), no: 57 (64%)
Depression at any point after the dissection	17 (19.1%)
Score above threshold for anxiety on HADS	13 (14.6%)
Score above threshold for depression on HADS	5 (5.6%)
Scores on SF-36 (*n* = 87)	Physical functioning: Mean: 91.7 (SD: 15.3) Role limitations due to physical health: Mean: 89.9 (SD: 23.8) Energy/fatigue: Mean: 63.7 (SD: 17.5) Emotional well-being: Mean: 79.2 (SD: 14.7) Social functioning: Mean: 88.7 (SD: 19.4) Pain: Mean: 86.5 (SD: 19.9) General health: Mean: 74.6 (SD: 16.1) Mean score across domains, all survivors: 82.8 Mean score across domains, PTSD: 69.8 Mean score across domains, anxiety: 75.5
All symptoms required for a PTSD present on PDS	10 (11.2%)
Symptom severity on PDS, regardless of presence of all formal symptoms	No-mild PTSD symptoms: 73 (82%) Mild–moderate PTSD symptoms: 8 (8.9%) Moderate–severe PTSD symptoms: 5 (5.6%) Severe PTSD symptoms: 3 (3.5%)
Score above threshold for abnormal fatigue on CIS	43 (48.3%)

**Table 3:** Exploration of differences between patients who met the criteria for PTSD at long term follow-up (*n* = 10) versus patients who did not (*n* = 79).

Variable	Total (*n* = 89)	Criteria for PTSD met		*p*-value
		Met (*n* = 10)	Not met (*n* = 79)	
Male/female (*n*, %)	50 (56%)/39 (44%)	5 (50%)/5 (50%)	45 (57%)/34 (43%)	*p* = 0.74
mRS before the dissection (median, IQR)	0 (0 to 0) missing = 1	0 (0 to 0)	0 (0 to 0) missing = 1	*p* = 0.68
Years of education (median, IQR)	15 (12.5 to 17.4) missing = 5	13.5 (11 to 16.3)	15 (12.5 to 17.6) missing = 5	*p* = 0.18
NIHSS score at admission (median, IQR)	0 (0 to 1) missing = 7	2 (0 to 4) missing = 1	0 (0 to 1) missing = 6	*p* = 0.43
Age at admission (median, IQR)	46 (37 to 52.5)	42.5 (36 to 50)	46 (37 to 53)	*p* = 0.5
Number of affected arteries (median, IQR)	1 (1 to 1)	1 (1 to 1.3)	1 (1 to 1)	*p* = 0.48
Dissection of right vertebral artery (no/yes) (*n*, %)	67 (75%)/22 (25%)	6 (60%)/4 (40%)	61 (77%)/18 (23%)	*p* = 0.26
Dissection of left vertebral artery (no/yes) (*n*, %)	74 (83%)/15 (17%)	8 (80%)/2 (20%)	66 (83%)/13 (16.5%)	*p* = 0.67
Dissection right carotid artery (no/yes) (*n*, %)	54 (61%)/35 (39%)	8 (80%)/2 (20%)	46 (58%)/33 (42%)	*p* = 0.3
Dissection left carotid artery (no/yes) (*n*, %)	58 (66%)/31 (35%)	5 (50%)/5 (50%)	53 (67%)/26 (33%)	*p* = 0.31
Occlusion of dissected artery (no/yes) (*n*, %)	67 (75%)/20 (23%) missing = 2	3 (30%)/6 (60%) missing = 1	64 (81%)/14 (18%) missing = 1	*p* = 0.004*
Stenosis of dissected artery (no/yes) (*n*, %)	40 (45%)/47 (53%) missing = 2	5 (50%)/4 (40%) missing = 1	35 (44%)/43 (54%) missing = 1	*p* = 0.73
Mural hematoma of dissected artery (no/yes) (*n*, %)	20 (23%)/64 (72%) missing = 5	3 (30%)/6 (60%) missing = 1	17 (22%)/58 (73%) missing = 4	*p* = 0.44
Hypertension at admission (no/yes) (*n*, %)	55 (62%)/31 (35%) missing = 3	8 (80%)/2 (20%)	47 (60)/29 (37%) missing = 3	*p* = 0.32
Hypercholesterolemia at admission (no/yes) (*n*, %)	75 (84%)/12 (13.5%) missing = 2	10 (100%)/0 (0%)	65 (82%)/12 (15%) missing = 2	*p* = 0.35
Diabetes at admission (no/yes) (*n*, %)	86 (97%)/1 (1%) missing = 2	10 (100%)/0 (0%)	76 (96%)/1 (1%) missing = 2	*p* = 1.0
Infection within 4 weeks before index event (no/yes) (*n*, %)	75 (84%)/13 (15%) missing = 1	9 (90%)/1 (10%)	66 (84%)/12 (15%) missing = 1	*p* = 1.0
Mechanical trigger event prior to dissection (no/yes) (*n*, %)	69 (78%)/19 (21%) missing = 1	8 (80%)/2 (20%)	61 (77%)/17 (22%) missing = 1	*p* = 1.0
Painful local symptoms at admission (no/yes) (*n*, %)	34 (38%)/55 (62%)	3 (30%)/7 (70%)	31 (39%)/48 (61%)	*p* = 0.74
Cervical pain at admission (no/yes) (*n*, %)	35 (39%)/54 (61%)	3 (30%)/7 (70%)	32 (40.5%)/47 (59.5%)	*p* = 0.73
Headache at admission (no/yes) (*n*, %)	23 (26%)/66 (74%)	2 (20%)/8 (80%)	21 (27%)/58 (73%)	*p* = 1.0
Cranial nerve palsy at admission (no/yes) (*n*, %)	68 (76%)/21 (24%)	9 (90%)/1 (10%)	59 (75%)/20 (25%)	*p* = 0.44
Horner syndrome at admission (no/yes) (*n*, %)	56 (63%)/32 (36%) missing = 1	7 (70%)/3 (30%)	49 (62%)/29 (37%) missing = 1	*p* = 0.74
Tinnitus at admission (no/yes) (*n*, %)	67 (75%)/21 (24%) missing = 1	5 (50%)/5 (50%)	62 (79%)/16 (20%) missing = 1	*p* = 0.05*
Stroke/infarct at admission (no/yes) (*n*, %)	50 (56%)/39 (44%)	3 (30%)/7 (70%)	47 (59.5%)/32 (40.5%)	*p* = 0.1
Transient Ischemic Attack at admission (no/yes) (*n*, %)	77 (86.5%)/12 (13.5%)	9 (90%)/1 (10%)	68 (86%)/11 (14%)	*p* = 1.0
Total monocular blindness at admission (no/yes) (*n*, %)	87 (98%)/2 (2%)	10 (100%)/0 (0%)	77 (97.5%)/2 (2.5%)	*p* = 1.0
Smoking at admission (no/yes) (*n*, %)	69 (78%)/17 (19%) missing = 3	4 (40%)/6 (60%)	65 (82%)/11 (14%) missing = 3	*p* = 0.003*
History of migraine without aura (no/yes) (*n*, %)	71 (80%)/16 (18%) missing = 2	10 (100%)/0 (0%)	61 (77%)/16 (20%) missing = 2	*p* = 0.2
History of migraine with aura (no/yes) (*n*, %)	81 (91%)/6 (7%) missing = 2	10 (100%)/0 (0%)	71 (90%)/6 (8%) missing = 2	*p* = 1.0
History of depression (no/yes) (*n*, %)	72 (81%)/14 (16%) missing = 3	7 (70%)/2 (20%) missing = 1	65 (82%)/12 (15%) missing = 2	*p* = 0.64
PDEQ score at admission (median, IQR)	15 (10 to 26.8)	23.5 (10.75 to 39.25)	15 (10 to 24)	*p* = 0.16
mRS after 3 months (median, IQR)	0,5 (0 to 1)	1 (0 to 2)	0 (0 to 1)	*p* = 0.1
Stroke after 3 months (*n*, %)	82 (92%)/4 (5%) missing = 3	8 (80%)/2 (20%)	74 (94%)/2 (2.5%) missing = 3	*p* = 0.07
Transient Ischemic Attack after 3 months (*n*, %)	85 (96%)/1 (1%) missing = 3	10 (100%)/0 (0%)	75 (95%)/1 (1%) missing = 3	*p* = 1.0
Horner syndrome after 3 months (*n*, %)	83 (93%)/3 (3%) missing = 3	10 (100%)/0 (0%)	73 (92%)/3 (4%) missing = 3	*p* = 1.0
New dissection after 3 months (*n*, %)	82 (92%)/4 (5%) missing = 3	8 (80%)/2 (20%)	74 (94%)/2 (3%) missing = 3	*p* = 0.07
Scores on SF-36 at LT-FU (median, IQR)	95 (90 to 100)	92.5 (72.5 to 100)	100 (90 to 100)	Physical functioning: *p* = 0.25
	100 (100 to 100)	100 (37.5 to 100)	100 (100 to 100)	Role limitations, physical health: *p* = 0.25
	100 (100 to 100)	100 (100 to 100)	100 (100 to 100)	Role limitations, emotional problems: *p* = 0.22
	70 (52.5 to 75)	50 (37.5 to 72.5)	70 (60 to 75)	Energy/fatigue: *p* = 0.03*
	84 (72 to 90)	66 (50 to 85)	84 (76 to 92)	Emotional well-being: *p* = 0.05*
	100 (75 to 100)	75 (50 to 100)	100 (88 to 100)	Social functioning: *p* = 0.03*
	100 (78 to 100)	78 (56.4 to 100)	100 (80 to 100)	Pain: *p* = 0.07
	80 (65 to 85)	65 (48.8 to 81.2)	80 (65 to 85)	General health: *p* = 0.05*
CIS score at long-term follow-up (median, IQR)	34 (34 to 38)	32 (28 to 34)	35 (30 to 38)	*p* = 0.09

*significant *p*-value.

### Anxiety, depression, and fatigue at long-term follow-up

32 (36%) participants reported feeling more anxious after the dissection compared to before (Table 2). The threshold for a probable anxiety diagnosis (8 points or above on the HADS) was met by 13 (14.6%) of the participants at long term follow-up (Table 2). Participants who reported a higher level of anxiety (as compared to prior to index CeAD) were more likely to report headache at admission (29 [91%] versus 36[65.5%]) and higher PDEQ score during the index event (median: 20.5 [IQR: 13.5 to 35] versus 13 [IQR: 10 to 20.3]). In addition, evidence was observed that participants who reported being more anxious after the CeAD had lower QoL-score on the following subdomains: role limitations due to emotional problems, energy/fatigue, emotional well-being, social functioning, pain and general health (Table 4). An independent *t*-test was performed as a sensitivity analysis to explore if there were significant differences in heightened anxiety occurrence between patients who participated in the long-term follow-up study more than 5 years after CeAD (38 patients) or less than 5 years after CeAD (51 patients). No significant differences were found (*p* = 0.475).

**Table 4:** Exploration of differences between patients who reported a higher level of anxiety at long term follow-up (*n* = 32) compared to patients who did not (*n* = 55), missing = 2.

Variable	Total (*n* = 87)	Higher level of anxiety reported at long term follow-up compared to before the dissection		*p*-value
		Yes (*n* = 32)	No (*n* = 55)	
Male/female (*n*, %)	49 (56%)/38 (44%)	18 (56%)/14 (44%)	31 (56%)/2 4 (44%)	*p* = 0.99
mRS before the dissection (median, IQR)	0 (0 to 0) missing = 1	0 (0 to 0)	0 (0 to 0) missing = 1	*p* = 0.05*
Years of education (median, IQR)	15 (12.5-17,4) missing = 3	16 (13–17.5) missing = 1	15 (12.25–17.25) missing = 2	*p* = 0.4
NIHSS score at admission (median, IQR)	0 (0 to 1) missing = 7	0 (0 to 1.5) missing = 3	0 (0 to 1) missing = 3	*p* = 0.8
Age at admission (median, IQR)	46 (37 to 52)	44.5 (36 to 52)	46 (38 to 53)	*p* = 0.9
Number of affected arteries (median, IQR)	1 (1 to 1)	1 (1 to 1)	1 (1 to 1)	*p* = 0.35
Dissection of right vertebral artery (no/yes) (*n*, %)	65 (75%)/22 (25%)	23 (72%)/9 (28%)	42 (76%)/13 (24%)	*p* = 0.64
Dissection of left vertebral artery (no/yes) (*n*, %)	72 (83%)/15 (17%)	29 (91%)/3 (9%)	43 (78%)/12 (22%)	*p* = 0.14
Dissection right carotid artery (no/yes) (*n*, %)	53 (61%)/34 (39%)	20 (62.5%)/12 (37.5%)	33 (60%)/22 (40%)	*p* = 0.82
Dissection left carotid artery (no/yes) (*n*, %)	57 (65.5%)/30 (34.5%)	17 (53%)/15 (47%)	40 (73%)/15 (27%)	*p* = 0.06
Occlusion of dissected artery (no/yes) (*n*, %)	66 (76%)/19 (22%) Missing = 2	21 (66%)/10 (31%) missing = 1	45 (82%)/9 (16%) missing = 1	*p* = 0.1
Stenosis of dissected artery (no/yes) (*n*, %)	39 (45%)/46 (53%) Missing = 2	14 (44%)/17 (53%) missing = 1	25 (46%)/29 (73%) missing = 1	*p* = 0.9
Mural hematoma of dissected artery (no/yes) (*n*, %)	19 (22%)/63 (72%) Missing = 5	10 (31%)/21 (66%) missing = 1	9 (16%)/42 (76%) missing = 4	*p* = 0.13
Hypertension at admission (no/yes) (*n*, %)	54 (62%)/30 (35%) Missing = 3	21 (66%)/10 (31%) missing = 1	33 (60%)/20 (36%) missing = 2	*p* = 0.61
Hypercholesterolemia at admission (no/yes) (*n*, %)	74 (85%)/11 (13%) Missing = 2	27 (84%)/5 (16%)	47 (86%)/6 (11%) missing = 2	*p* = 0.74
Diabetes at admission (no/yes) (*n*, %)	84 (97%)/1 (1%) Missing = 2	32 (100%)/0 (0%)	52 (95%)/1 (2%) missing = 2	*p* = 1.0
Infection within 4 weeks before index event (no/yes) (*n*, %)	73 (84%)/13 (15%) Missing = 1	27 (84%)/5 (16%)	46 (84%)/8 (15%) missing = 1	*p* = 0.92
Mechanical trigger event prior to dissection (no/yes) (*n*, %)	68 (78%)/18 (21%) Missing = 1	24 (75%)/8 (25%)	44 (80%)/10 (18%) missing = 1	*p* = 0.48
Painful local symptoms at admission (no/yes) (*n*, %)	33 (38%)/54 (62%)	12 (37.5%)/20 (62.5%)	21 (38%)/34 (62%)	*p* = 0.9
Cervical pain at admission (no/yes) (*n*, %)	35 (40%)/52 (60%)	16 (50%)/16 (50%)	19 (34.5%)/36 (65.5%)	*p* = 0.16
Headache at admission (no/yes) (*n*, %)	22 (25%)/65 (75%)	3 (9%)/29 (91%)	19 (34.5%)/36 (65.5%)	*p* = 0.009*
Cranial nerve palsy at admission (no/yes) (*n*, %)	67 (77%)/20 (23%)	27 (84%)/5 (16%)	40 (73%)/15 (27%)	*p* = 0.21
Horner syndrome at admission (no/yes) (*n*, %)	54 (62%)/32 (37%) Missing = 1	20 (62.5%)/12 (37.5%)	34 (62%)/20 (36%) missing = 1	*p* = 0.97
Tinnitus at admission (no/yes) (*n*, %)	65 (75%)/21 (24%) Missing = 1	21 (66%)/11 (34%)	44 (80%)/10 (18%) missing = 1	*p* = 0.1
Stroke/infarct at admission (no/yes) (*n*, %)	50 (57.5%)/37 (42.5%)	19 (59%)/13 (41%)	31 (56%)/24 (44%)	*p* = 0.78
Transient Ischemic Attack at admission (no/yes) (*n*, %)	76 (87%)/11 (13%)	26 (81%)/6 (19%)	50 (91%)/5 (9%)	*p* = 0.2
Total monocular blindness at admission (no/yes) (*n*, %)	85 (98%)/2 (2%)	30 (94%)/2 (6%)	55 (100%)/0 (0%)	*p* = 0.13
Smoking at admission (no/yes) (*n*, %)	68 (78%)/16 (18%) Missing = 3	25 (78%)/7 (22%)	43 (78%)/9 (16%) missing = 3	*p* = 0.61
History of migraine without aura (no/yes) (*n*, %)	69 (79%)/16 (18%) Missing = 2	27 (84%)/4 (13%) missing = 1	42 (76%)/12 (22%) missing = 1	*p* = 0.29
History of migraine with aura (no/yes) (*n*, %)	79 (91%)/6 (7%) Missing = 2	30 (94%)/1 (3%) missing = 1	49 (89%)/5 (9%) missing = 1	*p* = 0.30
History of depression (no/yes) (*n*, %)	14 (16%)/72 (83%) Missing = 1	4 (13%)/27 (84%) missing = 1	10 (18%)/45 (82%)	*p* = 0.52
PDEQ score at admission (median, IQR)	15 (10–26.8) missing = 1	20.5 (13.5–35)	13 (10–20.3) missing = 1	*p* = 0.003*
mRS after 3months (median, IQR)	0.5 (0 to 1) missing = 1	1 (0 to 1)	0.5 (0 to 1) missing = 1	*p* = 0.38
Stroke after 3 months (*n*, %)	80 (92%)/4 (5%) Missing = 3	30 (94%)/2 (6%)	50 (91%)/2 (4%) missing = 3	*p* = 0.62
Transient ischemic attack after 3 months (*n*, %)	83 (95%)/1 (1%) Missing = 3	32 (100%)/0 (0%)	51 (93%)/1 (2%) missing = 3	*p* = 0.43
Horner syndrome after 3 months (*n*, %)	81 (93%)/3 (3%) Missing = 3	31 (97%)/1 (3%)	50 (91%)/2 (4%) missing = 3	*p* = 1.0
New dissection after 3 months (*n*, %)	80 (92%)/4 (5%) Missing = 3	30 (94%)/2 (6%)	50 (91%)/2 (4%) missing = 3	*p* = 0.63
Scores on SF-36 at LT-FU (median, IQR)	95 (90 to 100)	95 (81.3 to 100)	100 (95 to 100)	Physical functioning: *p* = 0.1
	100 (100 to 100)	100 (75 to 100)	100 (100 to 100)	Role limitations, physical health: *p* = 0.09
	100 (100 to 100)	100 (67 to 100)	100 (100 to 100)	Role limitations, emotional problems: *p* = 0.01*
	70 (52.5 to 75)	60 (45 to 73.8)	70 (60 to 75)	Energy/fatigue: *p* = 0.02*
	84 (72 to 90)	76 (56 to 84)	88 (76 to 92)	emotional well-being: *p* < 0.001*
	100 (75 to 100)	94 (63 to 100)	100 (88 to 100)	Social functioning: *p* = 0.01*
	100 (78 to 100)	89 (57.6 to 100)	100 (90 to 100)	Pain: *p* = 0.04*
	80 (65 to 85)	70 (60 to 80)	80 (70 to 90)	General health: *p* = 0.01*
CIS score at long-term follow-up (median, IQR)	34 (34 to 38)	33 (30 to 38)	35 (30 to 38)	*p* = 0.97

*significant *p*-value.

17 (19.1%) participants reported experiencing depression at any point between the index event and long-term follow-up (Table 2), and the threshold for a probable depression diagnosis (8 points or above on the HADS) was met by 5 (5.6%) of the participants at long term follow-up (Table 2). 43 (48.3%) participants scored above threshold for abnormal fatigue on the CIS. No evidence was observed that scores above threshold for abnormal fatigue correlated with heightened levels of anxiety (*p* = 0.97, Table 4) and/or presence of PTSD (*p* = 0.09, Table 4). Thirty-nine (43.8%) participants still avoided and/or reduced physical activities as a direct result of the CeAD and participants still worked a mean of 3.4 h less weekly compared to before the index CeAD. There we no differences in QoL between male and female CeAD-survivors at long-term follow-up (Table 5).

**Table 5:** Comparison of differences in QoL between male and female patients at long term follow-up.

Variable	Total (*n* = 89)	Male versus female		*p*-value
		Male (*n* = 50)	Female (*n* = 39)	
Change in job hours at LT-FU (median, IQR)	0 (−4 to 0) missing = 16	0 (0 to 0) missing = 7	0 (−8 to 1) missing = 9	*p* = 0.71
Change in physical activity at LT-FU (median, IQR)	0 (−2 to 0.3) missing = 7	0 (−2 to 0.75) missing = 2	0 (−2.3 to 0.4) missing = 5	*p* = 0.92
Subjective health at LT-FU (0–100) (median, IQR)	90 (80 to 90) missing = 3	90 (80 to 90) missing = 1	90 (80 to 95) missing = 2	*p* = 0.27
Feeling more anxious after the dissection (no/yes) (*n*, %)	55 (62%)/32 (36%) missing = 2	31 (62%)/18 (36%) missing = 1	24 (61.5%)/14 (36%) missing = 1	*p* = 1.0
Depression at any point after the dissection (*n*, %)	69 (78%)/17 (19%) missing = 3	43 (86%)/6 (12%) missing = 1	26 (67%)/11 (28%) missing = 2	*p* = 0.04*
Anxiety score on HADS at LT-FU (median, IQR)	3 (1 to 6)	3 (1 to 7)	3 (2 to 5)	*p* = 0.68
Depression score on HADS at LT-FU (median, IQR)	1 (0 to 3)	1 (0 to 4.5)	1 (0 to 2)	*p* = 0.19
All symptoms of PTSD present on PDS at LT-FU (*n*, %)	79 (89%)/10 (11%)	45 (90%)/5 (10%)	34 (87%)/5 (13%)	*p* = 0.74
Symptom severity on the PDS at LT-FU (median, IQR)	3 (0 to 8)	3 (0 to 8)	3 (0 to 8)	*p* = 0.28
Scores on SF-36 at LT-FU (median, IQR)	95 (90 to 100)	100 (90 to 100)	95 (90 to 100)	Physical functioning: *p* = 0.36
	100 (100 to 100)	100 (100 to 100)	100 (100 to 100)	Role limitations, physical health: *p* = 0.49
	100 (100 to 100)	100 (100 to 100)	100 (100 to 100)	Role limitations, emotional problems: *p* = 0.99
	70 (52.5 to 75)	70 (53.8 to 80)	70 (50 to 75)	Energy/fatigue: *p* = 0.18
	84 (72 to 90)	84 (72 to 92)	84 (72 to 88)	emotional well-being: *p* = 0.15
	100 (75 to 100)	100 (88 to 100)	100 (75 to 100)	Social functioning: *p* = 0.19
	100 (78 to 100)	100 (80 to 100)	100 (68 to 100)	Pain: *p* = 0.43
	80 (65 to 85)	75 (63.8 to 85)	80 (65 to 85)	General health: *p* = 0.67
CIS score at long-term follow-up (median, IQR)	34 (30 to 38)	35 (29.8 to 38)	34 (30 to 38)	*p* = 0.77

*significant *p*-value.

### Quality of Life at long-term follow-up

The average QoL-score across all eight domains of QoL was 82.8 out of 100 for all patients, 69.8 for participants with PTSD and 75.5 for patients with heightened levels of anxiety (Table 2).

Figure 3 presents the scores from the SF-36 questionnaire. Participants were divided into three groups: Presence of PTSD (*n* = 10), heightened level of anxiety after CeAD (*n* = 32) and remaining CeAD patients (*n* = 57). The figure also presents normative SF-36 data from 9,332 healthy, age-matched (45 to 54 years) British adults averaged across the norms for males and females (18). Figure 3 illustrates that of those CeAD patients without increased levels of anxiety or who did not meet the criteria for PTSD on the PDS, none had reduced scores on any of the eight broad QOL-domains measured by the SF-36 compared to an age-matched normative sample. Evidence was found that patients who experienced increased levels of anxiety scored lower on six out of eight QOL-domains (role limitations due to emotional problems: *p* = 0.01, energy/fatigue: *p* = 0.02, emotional well-being: *p* < 0.001, social functioning: *p* = 0.01, pain: *p* = 0.04 and general health: *p* = 0.01) compared to patients who did not (Table 4). Similarly, evidence was found that participants who met the criteria for PTSD on the PDS scored lower on four out of eight QoL-domains (energy/fatigue: *p* = 0.03, emotional well-being: *p* = 0.05, social functioning: *p* = 0.03 and general health: *p* = 0.05) compared to CeAD patients who did not (Table 3).

**Figure 3:**
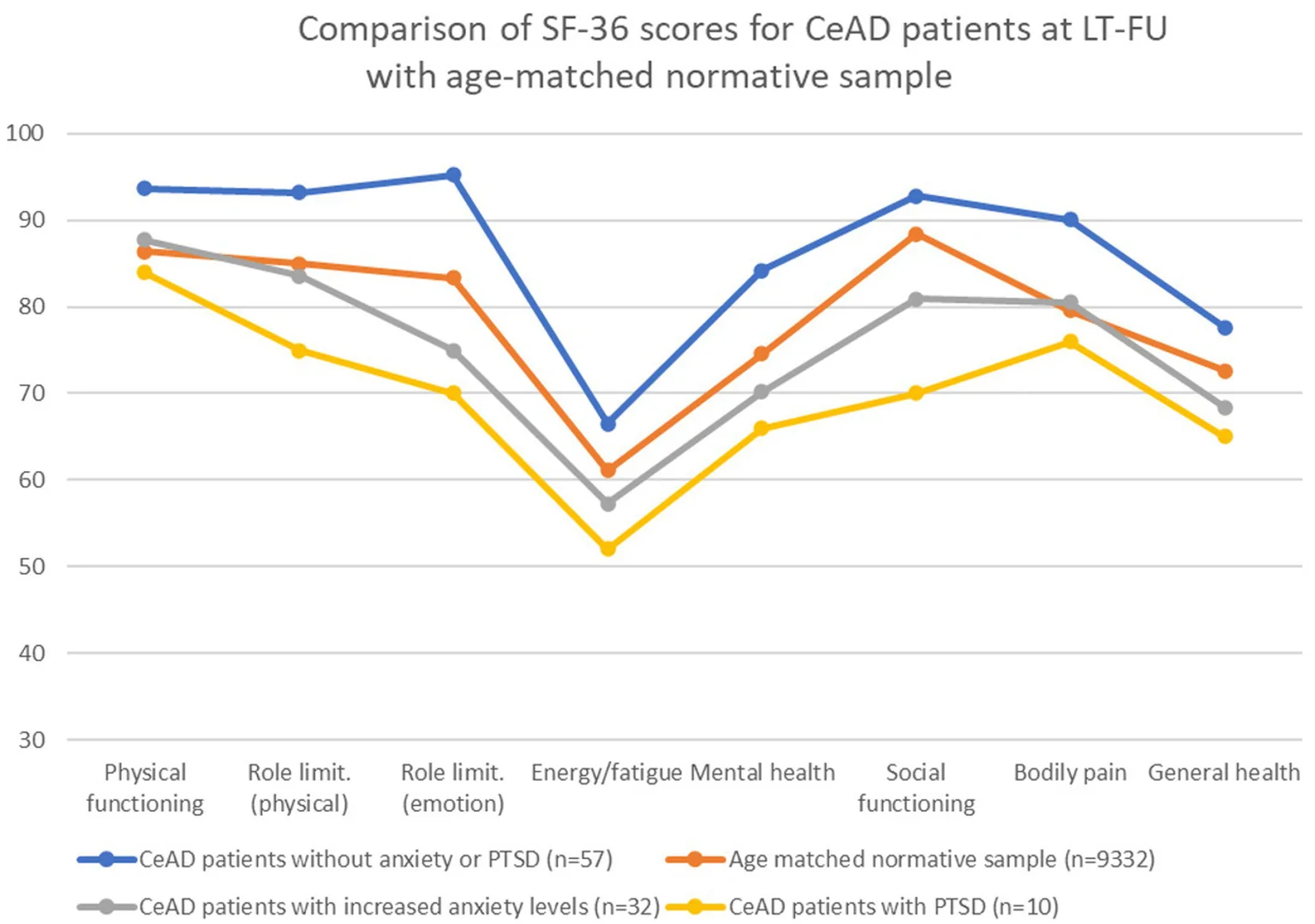
Visual representation of patient scoring patterns on SF-36: Comparison of patients who experienced increased levels of anxiety after CeAD (grey) and patients who did not (blue) with independent *t*-test found significantly reduced QOL on six out of eight QOL-domains (role limitations due to emotional problems: *p* = 0.01, energy/fatigue: *p* = 0.02, emotional well-being: *p* < 0.001, pain: *p* = 0.04, social functioning: *p* = 0.01 and general health: *p* = 0.01, see Table 4). Comparison of patients who met the formal criteria for a PTSD diagnosis (yellow) and CeAD patients who did not (blue) with independent t-test found significantly reduced QOL on four out of eight QoL-domains (energy/fatigue: *p* = 0.03, emotional well-being: *p* = 0.05, social functioning: *p* = 0.03 and general health: *p* = 0.05) compared to CeAD patients who did not (Table 3).

## Discussion

Among participants with a former cervical artery dissection more than a third of patients still reported anxiety at long term follow-up, and 11.2% of patients met the criteria for a PTSD diagnosis. Low QoL-scores in non-physical domains on the SF-36 co-occurred with heightened anxiety levels and PTSD, indicating that these mental health conditions could - at least partly—be responsible for impaired QoL after CeAD. Almost half of the participants still experienced severe mental fatigue at long-term follow-up despite excellent functional outcomes. More dissociation during the index event and a higher degree of cognitive complaints at long-term follow-up were associated with increased anxiety levels at long-term follow-up. Smoking and tinnitus at admission and occlusion of the dissected artery were associated with the presence of PTSD at long-term follow-up.

At long term follow-up, 18% of patients in the present study showed moderate to severe PTSD symptoms, while 11.2% of patients met the criteria for PTSD on the PDS. The frequency of patients meeting the PTSD criteria in our study was five times higher than the estimated 12-months PTSD-prevalence of 1.9% in the general Swiss population (20). An existing study on PTSD in 62 CeAD patients found that, during follow-up, 45.2% of patients met the criteria for PTSD (6). However, 15 of these patients were assessed within 1 month from the index event, and the remaining patients were assessed within a median of 18.9 months after the index event, which is a much shorter follow-up time than the patients in our cohort, who were followed up at least 1 year from the index event and on average 5.8 years after. Previous literature has shown that timing of PTSD-assessment is an important factor for diagnosis of PTSD (21). A recent meta-analysis of the prevalence of PTSD after all types of strokes estimated that 16.7% of all stroke survivors experienced PTSD symptoms (using any validated self-report instrument or diagnostic interview) 1 year after stroke (22) which is only slightly higher than the numbers obtained in this cohort of CeAD patients (11.2%). Interestingly, Tang et al. (2022) reported that the degree of disability after stroke predicted occurrence and severity of PTSD. Bearing this in mind, the occurrence of PTSD in our study sample is notable in the context of their excellent functional 3-month outcomes.

Significant dissociation predictive of an increased risk of developing PTSD was observed in 29.2% of CeAD patients during the initial admission. High scores on PDEQ have been shown to be associated with the development and severity of PTSD (14). It is therefore notable that high scores on the PDEQ did not significantly predict the presence of PTSD in the present study sample. Our findings could be confounded by the fact that patients with PTSD were compared with all other patients in the cohort, including those who experienced increased levels of anxiety, but who did not meet the criteria for PTSD on the PDS. Further, peritraumatic dissociation is common immediately after trauma but does not inevitably lead to PTSD, especially when protective factors are present (e.g., absence of prior psychiatric history—an inclusion criterion in our study) and our long median follow-up of 53 months may mean that some initial PTSD symptoms resolved over time.

Dissected artery occlusion is an important predictor of less favorable functional outcome in CeAD patients (23). Reasons for potential impact of dissected artery occlusion on higher PTSD numbers in CeAD patients remain elusive. One could speculate, that—even without subsequent cerebral ischemic events, the existence of an occluded main brain supplying artery is driving anxiety, distress and discomfort in CeAD patients and thus lead to PTSD. Likewise, patients with an occluded dissected artery might have been counselled about a higher perceived or potential risk of recurrent ischemic events. Awareness of this elevated risk—independent of whether a recurrent event actually occurs — may itself drive anxiety, distress and PTSD symptoms. While the presence of tinnitus at admission is known to predict a favorable functional outcome (24), it was associated with PTSD in the present study. Trauma-associated tinnitus is related to psychological distress, such as PTSD (25, 26), and the two often occur comorbidly (27). Nevertheless, in either condition it is unclear whether tinnitus causes PTSD or vice versa.

Smoking at the time of admission was significantly associated with PTSD, and in a systematic review, smokers have been reported to develop PTSD twice as often as nonsmokers (28). Therefore, our observation likely reflects the overall association between smoking and PTSD rather than a specific characteristic of dissection patients prone to PTSD.

In our study population, 14.6% of the CeAD-patients met the HADS criteria for anxiety at long-term follow-up - twice as high as the current global prevalence of anxiety in the general population at 7.3% (29). Comparing these numbers to the general stroke population is challenging since our data reflect point prevalence on average 6 years post index event, and no reports have been identified with data on anxiety at this timepoint after stroke. The estimated prevalence of anxiety within the first year after all types of strokes is 24.2% measured with rating scales and 18.7% by interview (30). Based on the well-recognized impact of anxiety on everyday functioning (31), it is highly likely that this condition significantly impacts daily life in this population with excellent physical outcome. The discrepancy between self-reported increase in anxiety (36%) and HADS-based probable anxiety diagnosis (14.6%) is likely to reflect different constructs being measured. The self-reported question captures a subjective, relative change in anxiety experience, whereas the HADS provides a standardized, cross-sectional assessment of current anxiety symptom severity against a clinical threshold. Many patients may perceive a meaningful increase in their anxiety levels that is nonetheless below the clinical threshold for a probable anxiety disorder. This is clinically relevant: Although these patients do not meet criteria for a probable anxiety disorder, elevated anxiety symptoms may still interfere with everyday functioning, including concentration, decision-making, social participation, and return to work. In a relatively young population such as CeAD survivors, who often recover well physically and are expected to resume work and family responsibilities, even modest but persistent anxiety symptoms may therefore have meaningful consequences and warrant clinical attention. It is advised that clinicians screen for anxiety even in CeAD-patients who do not meet formal diagnostic anxiety criteria.

In this study, 15.7% of the patients reported a history of depression prior to the index event, which is higher than the estimated lifetime prevalence of around 10% in the general population (32). 19,1% of patients had been diagnosed with depression at some point after the CeAD. This number is almost twice as high as the life-time prevalence of 10% in the general population (32). However, it is lower than the estimated prevalence of depression after stroke in general, which is approximately 30% (33, 34).

In the present study, CeAD-patients without increased scores for anxiety or PTSD did not experience diminished QoL compared to an age-matched normative sample on the SF-36 (Figure 3). CeAD patients with elevated scores for anxiety and PTSD primarily reported reduced QoL on non-physical domains on the SF-36, which is in line with earlier research in this patient group (7). This result indicates that increased scores for anxiety and the presence of PTSD could be partly responsible for the decrease in emotional and social QOL-domains experienced by many CeAD patients and underpins the importance of discovering and treating psychological sequelae after CeAD. In a recent study which examined QoL across all types of stroke, survivors obtained an average QoL-score of 62.1 across all eight domains on the SF-36 (35). This is lower than the average for CeAD patients, who obtained an average QoL-score of 82.8 for all patients, 69.8 for those with PTSD and 75.5 for those with heightened levels of anxiety. This is explained by the lower scores on measures of physical functioning after stroke in general compared to this CeAD cohort. Another study examined QoL specifically in young adults (<50 years) and found an average QoL-score of 72.3 across all eight domains on the SF-36 with the lowest scores in the areas of fatigue and role limitations due to physical symptoms (36). This indicates that a higher degree of impaired QoL can be explained by physical symptoms and limitations in other young stroke groups compared to CeAD patients, who overall have great functional outcomes.

Almost half of the CeAD patients in this cohort reported abnormal levels of fatigue at long-term follow-up, which is equivalent to numbers reported in stroke in general (37). This underpins the great challenge of mental fatigue as a long-term consequence of all types of stroke including CeAD.

It is notable that almost half of the CeAD patients still avoided and/or reduced physical activities as a direct result of the CeAD and still worked less (compared to prior to index CeAD) several years after the index event, even though most patients had a good physical and functional outcome. Research from patients with subarachnoid hemorrhages and cancer suggests that the patients’ subjective perceptions of the risks connected to their own illness are not necessarily aligned with the clinicians’ views (38, 39). Based on this, it is important that clinicians are aware of patients who are possibly reducing/avoiding otherwise normal and healthy work- and physical activities out of fear so they can accommodate worried patients and help correct unwarranted fears. Further, the observed mean reduction of 3.4 working hours per week represents approximately an 8–10% reduction in a standard full-time working week and is clinically and socioeconomically meaningful, particularly given the young age of this patient population. This underscores that the functional impact of CeAD extends well beyond what is captured by the mRS alone. These findings highlight the need for targeted vocational rehabilitation and counseling regarding safe activity levels in CeAD patients.

## Limitations and bias

First, given the exploratory nature of the study, conservative correction of *p*-values (such as Bonferroni correction) was not performed. Therefore, individual *p*-values should be interpreted with caution, and statistically significant associations should be considered strictly hypothesis-generating. Replication in independent, adequately powered samples is needed.

The timing of the long-term follow-up assessment relative to the index event was heterogeneous in this cohort, which consequently increases heterogeneity in the cohort in a variety of variables including time since index event, age, recall bias regarding variables relating to the index event collected at long-term follow-up etc. Furthermore, the single center design likely limits generalizability and due to the limited size of the cohort statistical analyses of subgroups are underpowered and explorative in nature.

At the time of study conduct, no published Swiss population-based SF-36 normative data were available. Therefore, UK normative data were used as the comparator, as they represent one of the largest European SF-36 reference datasets. However, cultural, linguistic, and healthcare-system differences between the UK and Switzerland may influence SF-36 scoring patterns, therefore no statistical comparison has been conducted between the current patient data and the UK normative SF-36 data. Rather, the normative data are used as a visual representation of QOL in a similar European country, and statistical analyses have only been conducted between CeAD-patients with and without PTSD and heightened anxiety, as is evident from Table 3, 4.

The Posttraumatic Diagnostic Scale (PDS) used in this study is based on DSM-IV criteria for PTSD. We acknowledge that the DSM-5 introduced revisions to the diagnostic criteria for PTSD, including changes to symptom clusters and the addition of new symptoms. The PDS was selected because it was the established and validated instrument available at the time of study initiation. The core symptom domains assessed by the PDS remain clinically relevant, and the impact of using DSM-IV-based criteria on PTSD prevalence estimates in the present study is uncertain but likely modest. However, a national sample of U. S. adults found that PTSD-prevalence was slightly lower using DSM-V rather than DSM-IV criteria, primarily due to the exclusion of nonaccidental, nonviolent deaths from Criterion A, and the added requirement of at least 1 active avoidance symptom in DSM-V (40). The change to criterion A is not relevant for this sample, but the new requirement of at least 1 active avoidance symptom could be. It is recommended that future studies use DSM-5-aligned instruments (e.g., PCL-5).

Every effort was done to increase participation and reduce selection bias, as described in the methods section. However, 59% of the eligible patients responded (89 of 151, see Figure 1) which makes some degree of responder bias likely, as non-responders may differ from responders in clinically important ways It is possible that patients with more severe fatigue, cognitive difficulties, anxiety, or PTSD symptoms may have lacked the energy or capacity to complete the questionnaires, potentially leading to an underestimation of symptom burden. Conversely, patients with high quality of life and few or no persisting symptoms may have felt less motivated to participate because they had largely moved on from the event, which could bias results in the opposite direction. As information on non-responders was limited, the direction and magnitude of any potential responder bias cannot be determined with certainty. We had no formal informed consent of the non-responders and thus could not include any data of these patients in our analyses to explore potential systematic differences between responders and non-responders. Reassuringly, however, all 151 potentially eligible patients screened for participation had equally excellent functional outcome at 3 months (as defined in the inclusion criteria of this study) indicating comparable baseline impairment. Furthermore, we excluded patients with an mRS of 2 or more at 3 months, as we were primarily interested in patients with excellent functional outcome and aimed to avoid confounding by functionally relevant motor or language impairments present at 3 months, and the mixed follow-up methods (postal/phone/email) possibly introduce heterogeneity. However, this pragmatic approach was necessary to maximize participation in a relatively rare condition. Importantly, all participants completed the same standardized questionnaires regardless of contact method.

Further, we did not systematically assess concurrent traumatic or stressful life events occurring between the index CeAD and follow-up. PTSD symptoms attributed to the CeAD may have been caused or exacerbated by subsequent unrelated events, and the absence of intercurrent event assessment may have inflated or attenuated the observed associations. It should be a priority for future prospective studies to include systematic assessment of intercurrent life events.

For the participants who missed one or a few questions in the questionnaires, the missing score was replaced with an average score calculated from the remaining relevant questions in the patient’s questionnaire. This method - mean imputation - is a simple but bias-inducing method that can attenuate variance and distort correlations. Multiple imputation was not considered an attractive alternative, as it most often relies on chained equations to estimate the missing based on the remaining data. If the sample size is small like the one in the current study, the imputation procedure cannot be expected to obtain a stable estimate of the missing data and hence will not converge. As such, mean imputation was chosen to ensure that we did not lose data from participants who wished to contribute but had inadvertently skipped a question.

Another central limitation of this study is that we did not account for other potentially traumatic or stressful life events that may have occurred in the interim between the CeAD and long-term follow-up, which increases the likelihood that PTSD-symptoms were caused by newer events unrelated to the CeAD.

The absence of a matched control group of individuals without CeAD is a further limitation of this study. Instead, published normative SF-36 data and general population prevalence estimates for PTSD, anxiety, and depression were used as reference points, which is a common approach in rare-disease research where assembling matched control groups may be logistically challenging. Future multicenter studies should aim to include age- and sex-matched controls to strengthen the interpretation of findings.

Finally, our findings possibly represent a conservative estimate of the true burden of psychological sequelae across all CeAD patients, as those with greater functional impairment—who likely are at even higher psychological risk—were excluded by design. This exclusion criterion may have led to an underestimation of the true psychological burden in all CeAD patients, and it is important to acknowledge that the present findings apply only to the subgroup of CeAD patients with excellent early functional recovery.

### Strengths

First, our study was based on a prospective CeAD registry comprising detailed sets of baseline variables, which could be analyzed alongside the long-term follow-up data. Second, to our knowledge, no other studies in CeAD patients exist, comprising equally longitudinal mental-health data in a CeAD-cohort and the here presented wide array of standardized questionnaires.

### Future perspectives

Comparison with QoL-scores in other groups of young stroke survivors indicate that physical symptoms explain impaired QoL to a higher degree in other types of stroke compared to CeAD patients. Earlier research in stroke suggests that certain personality traits - such as a high level of neuroticism and low level of openness - may impact the mental well-being of stroke survivors, both regarding overall QoL (41, 42), but also specifically regarding the development of PTSD (43), anxiety (44) and depression (45). As such, future studies should investigate the connection between personality traits and mental well-being after CeAD to identify patients with an increased risk of long-term mental health problems with the potential of modifying this risk through support or counseling. Furthermore, very recent research suggests a potential genetic component to the development of anxiety and depression after stroke (46)—a path worthwhile to pursue specifically in CeAD patients.

## Conclusion

Results from the present study suggest that psychosocial sequelae including PTSD, anxiety and impaired quality of life are frequent in patients with prior CeAD, many years after the index event and despite otherwise excellent functional outcome. As the present study is exploratory and hypothesis-generating in nature, further prospective, ideally interventional studies investigating psychological sequela in CeAD-patients are warranted before these variables are used in clinical risk stratification.

## Data Availability

Study data can be made available on reasonable request to the corresponding author. Such requests must be accompanied by detailed study proposals, a description of study objectives, and a statistical analysis plan. Additional material might be requested during the assessments of the study eligibility for data sharing, which must be approved by the corresponding author. Each request will be checked for compatibility with regulatory (ethics committee) requirements and with patient informed consent.
